# The Multifaceted Role of Pectin Methylesterase Inhibitors (PMEIs)

**DOI:** 10.3390/ijms19102878

**Published:** 2018-09-21

**Authors:** Alexandra Wormit, Björn Usadel

**Affiliations:** 1 Institute of Biology 1, Botany and Molecular Genetics, RWTH Aachen University, 52074 Aachen, Germany; usadel@bio1.rwth-aachen.de; 2 Bioeconomy Science Center (BioSC), Forschungszentrum Jülich, Wilhelm Johnen Straße, 52425 Jülich, Germany; 3 Institute for Bio- and Geosciences (IBG-2: Plant Sciences), Forschungszentrum Jülich, 52428 Jülich, Germany

**Keywords:** pectin methylesterase inhibitor (PMEI), pectin, homogalacturonan (HG), cell wall properties, degree of methylesterification (DM), stress, development, applications

## Abstract

Plant cell walls are complex and dynamic structures that play important roles in growth and development, as well as in response to stresses. Pectin is a major polysaccharide of cell walls rich in galacturonic acid (GalA). Homogalacturonan (HG) is considered the most abundant pectic polymer in plant cell walls and is partially methylesterified at the C6 atom of galacturonic acid. Its degree (and pattern) of methylation (DM) has been shown to affect biomechanical properties of the cell wall by making pectin susceptible for enzymatic de-polymerization and enabling gel formation. Pectin methylesterases (PMEs) catalyze the removal of methyl-groups from the HG backbone and their activity is modulated by a family of proteinaceous inhibitors known as pectin methylesterase inhibitors (PMEIs). As such, the interplay between PME and PMEI can be considered as a determinant of cell adhesion, cell wall porosity and elasticity, as well as a source of signaling molecules released upon cell wall stress. This review aims to highlight recent updates in our understanding of the PMEI gene family, their regulation and structure, interaction with PMEs, as well as their function in response to stress and during development.

## 1. Introduction

Plant cells are surrounded by a wall composed of interacting networks of polysaccharides, highly glycosylated proteins and other polymers. Plant cell walls are complex and highly dynamic structures, responding and adapting to normal processes of growth and development as well as to biotic and abiotic stresses. They have to fulfil several different functions: on one hand, they need to be flexible to allow fast and directional cell elongation (e.g., pollen tubes) and on the other hand they have to be rigid enough to resist the internal turgor pressure, to protect against pathogens and to provide structural and mechanical support for the upright growth of the whole organism.

The major carbohydrates of primary cell walls are cellulose, hemicellulose, and pectins. Cellulose and hemicellulose form a hydrogen-bonded network that is embedded in a gel-like pectic matrix. Pectin is a galacturonan-based polysaccharide containing five distinct subclasses that can be distinguished based on the structure of their backbones and the diversity of their side chains: homogalacturonan (HG), rhamnogalacturonan I and rhamnogalacturonan II (RG-II), xylogalacturonan and apiogalacturonan [1]. In dicots and non-graminaceous plants, pectins constitute about 35% of primary cell walls, whereas in grasses only 2–10% of primary walls are pectic polysaccharides [2]. Pectin has been shown to play roles controlling cell wall porosity [3], cell elongation [4], and cell adhesion [5] and constitutes an important factor in plant development (for reviews see [6,7]).

HG constitutes ~65% of pectin and is the most abundant pectic polysaccharide in primary cell walls [1]. It consists of an α-1,4-linked D-galacturonic acid (GalA) backbone, which is synthesized by HG galacturonosyl-transferases (GAUTs) from the nucleotide sugar uridine diphosphate (UDP)-GalA in the Golgi apparatus. So far, the galacturonosyl-transferase GAUT1 has been biochemically characterized in *Arabidopsis* [8,9] and a hetero-complex formation of two GAUT1 with one GAUT7 molecule has been shown responsible for retaining GAUT1 in the Golgi apparatus [10]. In addition, GAUT4 from *Arabidopsis thaliana*, switchgrass and poplar was shown to synthesize HG and downregulate of the gene reduced HG and RG-II in the cell wall [11]. When HG is secreted into the cell wall, it is highly methyl-esterified at the C6 atom of the GalA residue (~80% [2]) and a putative pectin methyltransferase conferring this methyl-esterification has been identified in the Golgi [12,13]. In addition, some GalA residues of HG carry acetyl groups at O2 or O3, which affect the physiochemical properties of HG in vitro [14].

**Figure 1:**
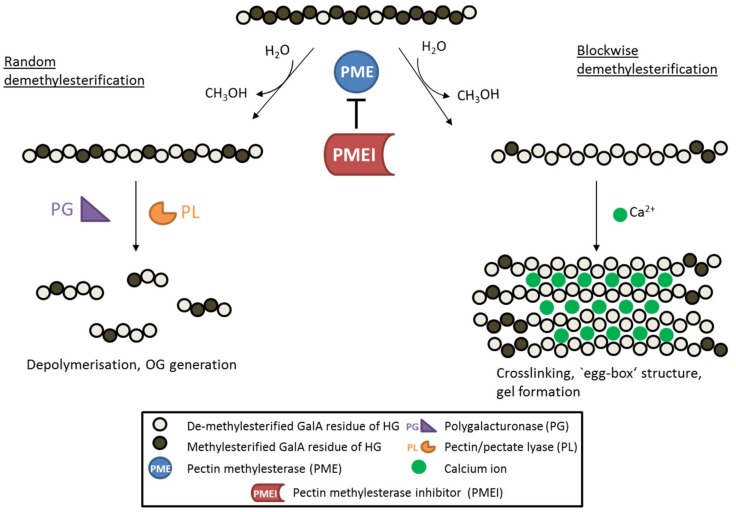
Schematic diagram showing the de-methylesterification of HG and the effects on its structure. HG is highly methylesterified when deposited into the cell wall. PMEs can de-methylesterify HG in a block-wise fashion, leading to several consecutive GalA residues without methylester groups. These HG backbones are negatively charged and can therefore form crosslinks with cations like calcium ions, leading to so called ‘egg-box‘ structures responsible for gel formation. On the other hand, PMEs can de-methylesterify single GalA residues leading to a random methylesterification pattern. Low-methylesterified HG is depolymerized by pectin-degrading enzymes such as polygalacturonases (PG) and pectin/pectate lyases (PL), which leads to the formation of oligogalacturonides (OG). PME activity is inhibited by its proteinaceous inhibitor PMEI.

Once incorporated into the cell wall, HG is further selectively modified and the pattern and degree of these modifications affect pectin hydrolysis and properties such as pH, charge and crosslinking. Pectin acetyl esterases catalyze de-acetylation and the release of acetate from the cell wall, causing changes in cell wall mechanics and developmental aberrations [15,16]. Pectin methyl esterases (PMEs) catalyze the specific de-methylesterification of HG, releasing methanol and protons, and creating negatively charged carboxyl groups in the process (Figure 1). The degree and pattern of methylesterification of HG determine the biomechanical properties of the cell wall. When several consecutive GalA residues are de-methylesterified (block-wise de-methylesterification), the negatively charged carboxyl groups can form calcium bonds with other HG molecules, leading to so-called ‘egg-box’ structures that underlie the formation of pectin gels [17]. De-methylesterified, calcium cross-linked HG increased the amount of bound water maintaining wall hydration [18] and the hydration state was shown to affect biomechanical properties of the cell wall, such as its rigidity [19]. In addition, the strength of pectin gels is highly dependent on the amount of free calcium ions in the apoplast, as stiffness of the gel is reduced by disassociation of calcium crosslinks [20]. On the other hand, partially de-methylesterified HG (random or block-wise de-methylesterification) can become a target for pectin-degrading enzymes such as polygalacturonases and pectate/pectin lyases.

The degree of methylesterification (DM) of HG, which is controlled by the activity of large PME families, has vast consequences on the mechanical properties of the cell wall [21], affecting developmental processes such as stomata opening [22,23], cell adhesion [5], organ initiation [24] and anisotropic cell growth [25]. In addition, hydrolysis of partially de-methylesterified HG can lead to the formation of signaling molecules (oligogalacturonides), for instance, during plant-pathogen interactions [26,27]. Consequently, PME activity is tightly regulated at: (a) the transcriptional level [28], (b) by protein processing [1] and degradation [29], (c) by the pH of the cell wall environment [6,30], and (d) by endogenous inhibitor proteins called pectin methylesterase inhibitors (PMEI, Figure 1) [31,32]. The first PMEI was identified in kiwi fruit (*Actinidia deliciosa* [33]) and to date several PMEIs have been investigated in different plant species. This review therefore aims to give a comprehensive overview on the current knowledge about PMEIs and their various roles in plant development and stress response.

## 2. PMEI Occurrence and Regulation

PMEIs belong to large multigene families, containing almost as many members as *PME* genes in several plant species [34]. The PMEI proteins first appeared in mosses (*Physcomitrella patens*), which coincided with the appearance of pectin in cell walls [34]. In *Arabidopsis*, 71 putative *PMEI* genes have been identified in silico (not including *proPME* genes containing PMEI domains) [34], compared to 100 open reading frames (ORFs) in *Brassica campestris* [35] and 97 putative *PMEI* genes in *Brassica rapa* [36]. Pinzón-Latorre and Deyholos [37] listed 95 PMEI ORFs in flax (*Linum usitatissimum*), whereas in poplar (*Populus trichocarpa*), only 54 genes have been annotated [34]. In monocots, the PMEI families generally contain fewer isoforms. For example, in rice (*Oryza sativa*), 49 ORFs were annotated as putative PMEIs [38] compared to 37 genes in *Sorghum bicolor* [34] and 38 putative PMEI members in *Brachypodium distachyon* [1]. The smaller family size in grasses is likely due to differences in the structure of cell wall polysaccharides, with pectins being less abundant and less methylesterified in graminaceous species [2,28]. Gene family synteny and phylogenetic analyses suggest that the expansion of the PMEI families in several angiosperm species is due to whole genome duplication and tandem duplication events during evolution [34,35,37].

**Figure 2:**
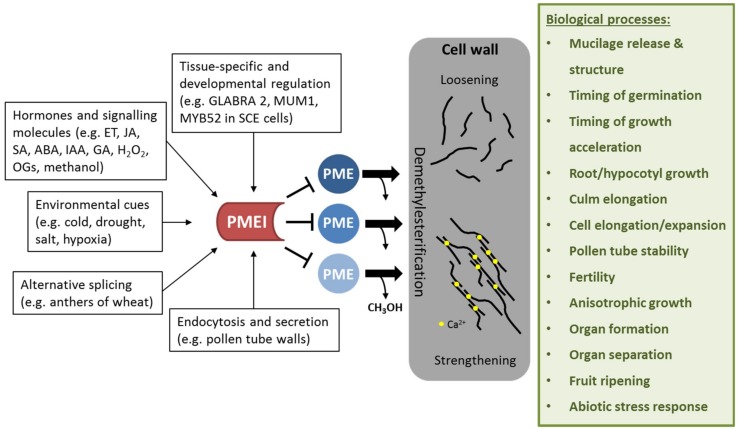
Effect of PMEI regulation on cell wall properties and biological processes affected by PMEI manipulation. *PMEI*s are transcriptionally regulated in a tissue-specific and development-dependent manner. Several plant hormones and signaling molecules as well as environmental stresses can activate *PMEI* gene expression. Alternative splicing and directed endocytosis and secretion regulate the level of active PMEIs in the cell wall. PMEI can inhibit several PME enzymes, thereby regulating de-methylesterification of HG. This in turn modulates cell wall properties such as loosening or strengthening, which is required for several biological processes.

PMEIs are crucial factors in regulating the DM of HG, which has tremendous effects on cell wall mechanics and affects many biological processes (Figure 2).

Therefore, it is not surprising that their expression is highly coordinated with other pectin modifying enzymes, such as PMEs [28]. Transcriptional analyses (using microarrays, RNAseq, qRT-PCR) showed that *PMEI* expression is spatially and developmentally controlled in various species (*Brassica rapa* [36], *Brassica campestris* [39], *Arabidopsis* [40], grapevine [41], wheat [42], rice [38], and flax [37,43]). Cluster analysis for a fraction of *PME* and *PMEI* genes already identified expression clusters specific for e.g., seed coat, endosperm, pollen, shoot apex in *Arabidopsis* [1]. For example, *AtPMEI6* and *AtPMEI14* are specifically expressed in seed coat epidermal cells and their expression is modulated by the transcriptional regulators GLABRA2 and MUCILAGE-MODIFIED 1 (MUM1), known to be involved in seed coat differentiation and mucilage production [44]. In addition, the R2R3-MYB (myeloblastosis) transcription factor MYB52 has been identified as a negative regulator of pectin de-methylesterification in seed coat mucilage due to its control of *AtPMEI6* and *AtPMEI14* expression [45].

Hormones are also involved in regulation of *PMEI* expression in several species [38,46,47,48,49]. For example, transcriptional activation of *PMEI* was shown to be ethylene-dependent during the ripening process of banana fruits [46], whereas a wheat *PMEI* was inducible by salicylic and jasmonic acid as well as hydrogen peroxide, indicating a role in defense responses [48]. Moreover, post-transcriptional regulation has also been indicated for PMEIs. Rocchi et al. [42] showed that two *PMEI* genes from durum wheat undergo intron retention. Mature transcripts were predominantly found in floral organs, indicating a role in flower development and in particular, anther and pollen development.

In pollen tubes, dynamic pectin metabolism is the key to polar growth and concurrently, different PME and PMEI isoforms are highly expressed in this cell type. By immuno-labelling, it was shown that HG with a low DM is found at the flanks of the pollen tube, whereas methylesterified pectin is predominantly present at the growing tip region where wall extensibility is required for cell growth [50,51]. This transition from apical to distal pectin epitopes seems to be correlated with an increase in cell wall rigidity and decrease in visco-elasticity. The maintenance of the distribution of esterified and de-esterified HG is ensured by a dedicated mechanism involving localized secretion of specific PMEI proteins [52]. The pectin methylesterase AtPPME1 is distributed evenly in the cell wall of the pollen tube. In contrast, the pollen-specific AtPMEI2, which interacts with AtPPME1, is exclusively localized at the cell wall of the pollen tube tip. This specific localization was shown to be dependent on endocytic internalization of AtPMEI2 at the flanks of the pollen tube. Therefore, protein secretion and endocytosis seems to be an efficient mechanism for regulating DM of pectin in a temporally and spatially controlled manner in pollen tubes. It remains to be seen if other PMEIs in other plant tissues are also regulated by such a mechanism.

In general, the large number of PMEI family members with distinct expression and regulation patterns could suggest the existence of dedicated interaction pairs required for localized modifications of HG methylesterification during development. Additionally, this could indicate different inhibitory activities of PMEIs depending on the cell wall environment and/or different specificities for target PMEs to guarantee a development- and stress-dependent adjustment of cell wall mechanics.

## 3. PME Inhibitor Structure and Interaction with Pectin Esterases

PME inhibitors and invertase inhibitors belong to the same protein family (PF04043, http://pfam.xfam.org/family/PF04043), although their respective target proteins are part of different metabolite pathways. The overall sequence identity between PMEIs and invertase inhibitors is only moderate (20–30%), but their three-dimensional structure is highly similar [53]. As shown by crystallographic approaches, PMEIs consist of four long α-helices arranged in an up-down-up-down topology, forming a four-helix bundle. They contain an N-terminal signal peptide responsible for extracellular targeting and four highly conserved cysteine residues, which are involved in the formation of two disulfide (S-S) bridges essential for the maintenance of the secondary structure of the protein [54,55]. One S-S bridge connects helices α2 and α3; the second S-S bridge is located in a fifth, N-terminal anchor region consisting of short distorted helices [56].

Interaction studies performed in vitro at different pH values (pH 5.5, 7.5 & 8.5; pH 7.5; pH 5.5 & 7.5; pH 6.5, 7.5 & 8.5) indicated formation of a stoichiometric 1:1 complex of PMEIs with endogenous PMEs but also with enzymes from other species [41,53,56,57]. For example, Jolie et al. [58] showed that purified PMEI from kiwi can bind to PMEs from carrot and broccoli (at pH 6.5), whereas tomato and *Arabidopsis* PMEIs can interact and inhibit PMEs from orange (at pH 7.5 and pH 7, respectively) [30,59]. On the other hand, plant PMEIs do not seem capable of inhibiting fungal or bacterial PMEs, as was shown for PMEI1 and PMEI2 from *Arabidopsis,* which did not affect PME activity of *Botrytis cinerea* [26]. Similarly, Reca et al. [59] showed that purified tomato PMEI only inhibited plant PMEs but was not active against PME from *Erwinia chrysantemi*. An explanation for this lack of inhibition might be that residues critical for PME-PMEI interaction are not conserved in fungal and bacterial PMEs [56].

The three-dimensional structure of *Arabidopsis* and kiwi PMEI interacting with purified PME from tomato revealed that the inhibitor covers the putative active site of the enzyme, thus preventing substrate binding [53]. The N-terminal anchor region interacts with the C-terminus of PME, potentially supporting the structural stability. The complex formation of PMEI with PME has been shown to be pH sensitive [60]. A detailed study on kiwi PMEI indicated that PMEI-PME complex formation in vitro was reduced at pH values above 6.0, due to reversible conformation changes in the 3D structure of the PMEI protein. At pH levels above 7.5, irreversible changes in PMEI secondary structure occur, such as cleavage of the S-S bridges, inducing helix instability. Once the PMEI-PME complex is formed, it is rather stable and only dissociates under extreme pH conditions (such as pH 10), indicating that interaction with the endogenous PME enzyme protects the inhibitor from conformational changes. Different PMEIs have been shown to function at different pH values; Nguyen et al. [61] analyzed a PMEI of rice (OsPMEI28), which had a highest inhibitor activity against commercial PME at pH 8.5, whereas complex formation between PME3 and PMEI7 of *Arabidopsis* was highly sensitive to pH variation between 5 and 7 [62]. The observed sensitivity was the result of changes in the protonation of amino acid residues at the interface of the two proteins, which caused residues to shift between inter- and intra-molecular interactions. These results indicate that the prevalent cell wall environment, i.e., the pH of the cell wall, can affect the activity of PMEIs, thereby fine-tuning the DM of pectic polysaccharides.

It was also shown that one PMEI protein can bind and inhibit several endogenous PME enzymes [39,56], indicating a complex mechanism for regulation of PME activity. For example, key residues involved in the interaction between the inhibitors AtPMEI4 or AtPMEI9 and AtPME3 were identified by computational approaches and indicated that functional diversity between the two PMEIs leads to distinct consequences on pollen tube elongation [63]. However, the existence of strictly specific PMEI-PME pairs cannot be excluded at this time.

## 4. Role of PMEIs in Stress

The cell wall is the first barrier against pathogens and maintenance of cell wall integrity plays an important role in pathogen defense [64]. For many bacteria and fungi, secretion of cell wall degrading enzymes that hydrolyze pectin is an important component for successfully infecting a plant [65,66,67,68]. Conversely, inactivation of pectin degrading enzymes in pathogens can reduce their virulence on host plants [69,70,71,72]. Plant PME activity and the level of pectin methylesterification are highly regulated by pathogens during an infection process and an increase in PME activity is correlated with reduced DM of pectin in *Arabidopsis* during infection with different pathogens [67,68,73]. In addition, degradation of HG by pathogenic polygalacturonases releases de-esterified pectin fragments or oligogalacturonides (OGs) which in turn act as damage-associated molecular patterns (DAMPS [74]). Several studies demonstrated that OGs are sensed by specific plant receptors (wall-associated kinase, WAK) and can activate a stress response by the plant [75,76]. It has been shown that the DM of OGs influences the strength of the elicited response [27,77]. Furthermore, PME activity on HG results in release of methanol, a signaling molecule that is involved in priming of (neighboring) plants and able to retard bacterial growth [78].

Constitutive expression of the *PMEI* genes *AtPMEI-1* and *AtPMEI-2* in *Arabidopsis* reduced the PME activity and resulted in an increase in DM of HG [26]. The transgenic mutants were more resistant to the necrotrophic fungus *Botrytis cinerea*, which was not due to inhibition of fungal PME but rather to an impaired fungal growth on methylesterified pectin. Further *PMEI* genes, *AtPMEI10*, *AtPMEI11* and *AtPMEI12*, were identified as upregulated in response to *B. cinerea* infection [47]. Genetic studies indicated that AtPMEI expression is dependent on jasmonic acid and ethylene signaling but only AtPMEI11 can be induced by OGs [47]. The *pmei10*, *pmei11* and *pmei12* mutants showed increased PME activity, decreased DM of pectin and increased lesion formation upon *B. cinerea* infection. This indicates that plants modulate PME activity by expressing PMEIs in response to pathogen attack.

Similarly, transgenic wheat lines expressing the *PMEI* from kiwi fruit also showed a reduction of disease symptoms caused by two fungal pathogens, *Fusarium gramineum* and *Bipolaris sorokiniana* [79]. This was related to reduced fungal growth on pectin with high DM and the activity of fungal PG to hydrolyze the plant pectin was impaired. In addition, the transgenic wheat plants showed less disease symptoms upon infection with the biotrophic fungal pathogen *Claviceps purpurea* [80]. This also shows the importance of pectin (and its methylesterification status) in grass species, despite its low abundance [2]. In barley (*Hordeum vulgare*), a family of putative pectin esterase inhibitors (PEIs) has been associated with a resistance gene (*Rrs2* locus), which is involved in defense against *Rhynchosporium commune* which causes leaf blotch [81]. Overexpression of *HvPEI4* led to an improvement of the resistance, but none of the candidate genes alone caused a high increase in resistance level. In a recent study, a PME inhibitor from cotton (*Gossypium hirsutum*) was identified as being involved in the defense response to the fungus *Verticilium dahliae* [39]. *GhPMEI3* expression in *Arabidopsis* led to reduced disease symptoms upon infection with *V. dahlia* and silencing of *GhPMEI3* in cotton resulted in an increased susceptibility to the fungus.

**Figure 3:**
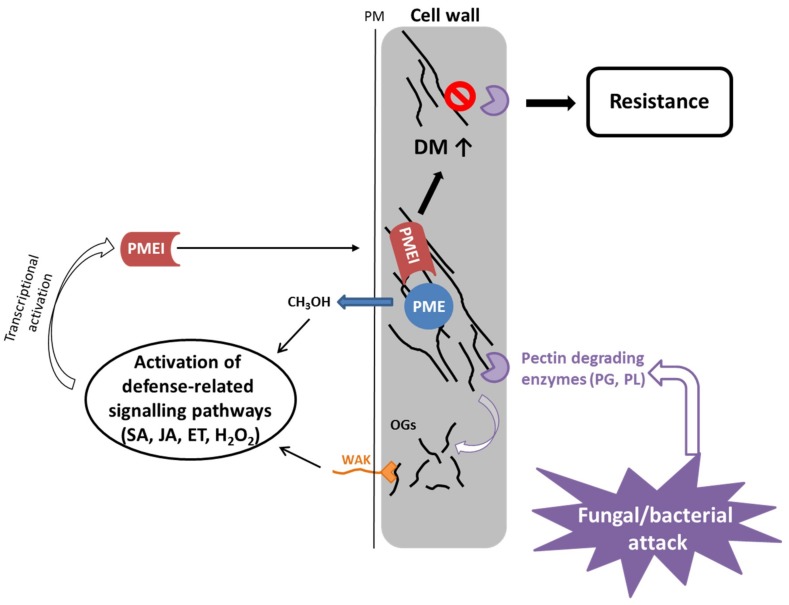
PMEIs modulate PME activity and DM of pectin in response to fungal and bacterial attack. Bacteria and fungi secrete pectin degrading enzymes, like polygalacturonases and pectate/pectin lyases, upon infection. PME activity leads to release of methanol, which serves as an alarm signal, activating the expression of pathogen-related PMEIs. Similarly, OGs, which are generated by degradation of pectin by fungal/bacterial PGs and PLs, serve as DAMPs, leading also to activation of defense related signaling pathways, which have been shown to activate *PMEI* gene expression. PMEIs inhibit PME activity leading to a higher degree of methylesterification of pectin, which contributes to resistance to fungal/bacterial enzymes resulting in less disease symptoms.

Based on current evidence, the proposed role of PMEI in the immune response is the dynamic modulation of the PME activity (Figure 3). The degree of methylesterification determines the susceptibility of the plant cell wall to the pectin-degrading enzymes of the pathogen [82]. Plant PME activity generates methanol as an alarm signal of the damaged self that can regulate the transcription of pathogen-related *PMEI* genes either directly (e.g., *AtPMEI11* [47]) or via modulation of DAMP or defense hormone signaling (*AtPMEI10*, *AtPMEI11*, *AtPMEI12* [47]). OGs are released by partial hydrolysis of HG by fungal/bacterial polygalacturonases. They serve as DAMPs and bind to WAKs at the plasmamembrane, leading to activation of plant immune signaling, which induces classic defense responses. The DM of OGs can influence the intensity of the defense response. PMEIs (e.g., *AtPMEI10*, *AtPMEI11*, *AtPMEI12* [47]) are transcriptionally upregulated by defense-related signaling pathways including jasmonic acid and ethylene, and inhibit plant PMEs leading to a higher DM of pectin, which makes it less susceptible to being broken down by fungal cell wall degrading enzymes.

PMEIs have also been implicated to play a role in defense against bacteria. *CaPMEI1* from pepper (*Capsicum annuum*) was identified in pepper leaves infected with *Xanthomonas campestris* pv. *vesicatoria* (*Xcv*) [83]. *Xcv* secretes plant cell wall degrading enzymes like pectate lyases and polygalacturonases upon infection. *CaPMEI1* was transcriptionally upregulated by different biotic stress signals (e.g., salicylic acid, jasmonic acid, ethylene, H_2_O_2_) and consequently, silenced pepper plants exhibited an increased susceptibility to *Xcv*. Conversely, overexpression of *CaPMEI1* in *Arabidopsis* conferred an enhanced resistance to *Pseudomonas syringae* but not to the biotrophic fungus *Hyaloperonospora parasitica*.

Little is known about the role of PMEIs in viral infections. In tobacco, plant PMEs have been shown to be involved in tobacco mosaic virus movement between host cells via interaction with the viral movement protein [84]. Accordingly, overexpression of *PMEIs* in tobacco and *Arabidopsis* counteracted the action of PMEs, leading to increased resistance to tobacco mosaic virus and turnip vein clearing virus infection respectively [85]. This was characterized by the reduced or delayed movement of the viruses and decreased disease symptoms. It is suggested that *PMEI* reduces viral spreading via limiting viral-induced PME activity and by hindering the enlargement of plasmodesmata.

Plants respond to abiotic stresses, e.g., water deficit, salt stress, and temperature extremes, with changes in cell wall architecture, although available literature is more focused on transcriptomic data rather than biochemical experiments [86,87]. PMEIs are also involved in the plants’ response to changing environmental conditions, however; only little is known about their molecular role. *CaPMEI1* from pepper is transcriptionally induced by cold treatment, drought stress, as well as the plant stress hormone abscisic acid and hydrogen peroxide [83]. *Arabidopsis* lines overexpressing *CaPMEI1* exhibited an increased tolerance to water stress, as shown by improved germination rate and seedling root growth in the presence of high mannitol concentrations as compared to control plants. In addition, a reduced transpiration rate was accompanied by less withering upon drought stress, and improved tolerance to oxidative stress caused by methyl viologen [83].

Several studies using expression analysis have revealed the functional importance of PMEIs in response to abiotic stresses. RT-PCR analysis of the wheat *TaPMEI* showed the responsiveness of the gene to H_2_O_2_ stimulus, salinity, water stress (caused by polyethylene glycol) and abscisic acid treatment [48]. Time course analysis further indicated a specificity of the transcriptional response based on the investigated tissue (leaf, stem and root). In silico meta expression analysis using Affymetrics array data for rice [38] revealed the importance for several *PMEI* family members in diverse stress responses. Drought stress, salt stress, cold and anaerobic conditions (during germination) were analyzed, and showed specific transcriptional regulations of *PMEI* genes [38]. Similarly, in *Arabidopsis,* the effect of hormone and stress treatments on the expression of *PMEI* family members was analyzed in silico. [35]. The microarray data analysis of the *PMEI* family showed that several *PMEI* are differentially expressed in response to abscisic acid, gibberellic acid, auxin and methyl jasmonate treatment, and many are involved in cold, drought, heat, oxidation, salt, and wounding regulation in *Arabidopsis* [35]. In the same study, a *cis*-element analysis of promotor regions of the *Brassica campestris* ssp. *chinensis PMEI* gene family also indicated an involvement of several *PMEI* genes in environmental stress responses. For example, of the 100 *PMEI* genes in *Brassica campestris*, 38 *BcPMEI* promotors contained both abscisic acid and auxin (indole-3-acetic acid) related *cis*-elements, 63 and 35 promotors contained heat stress elements and low-temperature response elements, respectively. Additionally, MYB binding sites involved in drought stress were found in 58 *PMEI* promotors [35]. However, these in silico results will have to be validated by experimental data.

An *Arabidopsis* mutant with reduced expression of a *PMEI* gene (At1g62760) exhibited reduced sensitivity to salt stress [88]. Enhanced root growth, higher fresh weight and less chlorosis and necrosis upon NaCl treatment were accompanied with reduced salt stress signaling in the mutant, suggesting this PMEI as a negative regulator of salinity tolerance. In contrast, overexpression of *AtPMEI13* (At5g62360) in *Arabidopsis* also led to increased salt tolerance, as transgenic lines showed higher rates of germination, root growth, and survival under salinity conditions as compared to wild type plants [89]. This phenotype was also observed in plants overexpressing the closest homolog *CbPMEI1* from *Chorispora bungeana*, an alpine plant highly tolerant to chilling and freezing stress. Both genes were repressed by salt stress and abscisic acid but were induced by cold, suggesting distinct roles of the genes in freezing and salinity tolerance. The transgenic overexpression plants showed decreased freezing tolerance; however, they exhibited increased root growth under cold conditions. Plants increase pectin levels in the cell wall under low temperatures [90] probably to reduce cell wall porosity and increase cell adhesion. It was previously reported that DM of pectin increases during cold acclimation in pea [91]. In contrast, loss of AtPME41 in *Arabidopsis* by a transfer DNA insertion caused an increase in freezing sensitivity [92]. Since pectins are important under low temperature conditions, PMEI activity could play a role in balancing the trade-off between freezing tolerance and growth by modifying the mechanical properties of cell walls.

## 5. Role of PMEIs in Development

PME-mediated changes in methyl-esterification of HG have been shown to affect various vegetative and reproductive stages of plant development (for reviews, see [6,7]). Therefore, we aim to give an overview of the latest results of molecular studies on PMEIs in plant development.

### 5.1. PMEI Function in Seeds

Upon hydration, mature *Arabidopsis* seeds rapidly extrude a gelatinous substance from seed coat epidermal (SCE) cells called mucilage, which is mainly composed of cell wall polysaccharides [93]. This layer has adhesive properties and possibly serves as a water-reservoir, which might facilitate seed germination, although it has been shown that mucilage production is not necessary for germination or plant fitness in the laboratory [94,95]. The mucilage is mainly composed of pectin, RG-I and small amounts of HG, but hemicellulose and cellulose have also been identified as minor components, as well as arabinogalactan proteins [96]. Several studies provided evidence that the DM of HG determines mucilage properties. For example, seven *PME* genes are specifically expressed in *Arabidopsis* seeds and the *pme58* mutant caused an altered pectin distribution in the mucilage [97]. Additionally, an E3 ubiquitin ligase, FLYING SAUCER1, was proposed to regulate the DM and a mutation lead to reduced mucilage extrusion and increased mucilage adherence [29]. Finally, the subtilase *atsbt1.7* mutant didn’t show significant sugar compositional changes in its mucilage but a decrease in mucilage methylation, which was correlated with an increased PME activity [98].

More direct evidence was obtained by investigating the role of *PMEI6*, which was shown to be essential for proper mucilage release [44]. In three *pmei6* mutants, the outer primary cell wall of the SCE cells did not rupture into fragments but remained attached, which caused a delay in mucilage extrusion. This was accompanied by a lack of methylesterified HG (detected by antibodies) and reduced methanol release in the mucilage, as well as increased PME activity in seeds. The reduction in methylesterification in the outer primary cell wall of the three *pmei6* mutants could possibly lead to more abundant Ca^2+^ cross-linking of HG, which causes stronger attachment between adjoining cell walls and therefore impairs release of the mucilage. Further evidence indicates that PMEI14 is also involved in regulating pectin methyl-esterification in seed coat mucilage and that *PMEI14* together with *PMEI6* and *ATSBT1.7* are likely transcriptionally controlled by MYB52 [45]. Knock out mutants of *PMEI14* had an increased PME activity in seed mucilage but not in demucilated seeds. A reduced level of methylesterification of the mucilage was accompanied by an increased amount of calcium cross-linked HG detected by immuno-labelling. These studies show that PMEI6 function affects both seed coat mucilage and the primary SCE cell wall, and is necessary for proper mucilage release. In contrast, PMEI14 function is confined to inhibiting PME activity, specifically in the seed coat mucilage, potentially influencing pectin structure.

PME activity has also been shown to be tightly controlled during germination in *Arabidopsis* and garden cress (*Lepidium sativum*) [99,100], probably leading to cell wall weakening to allow radicle emergence. A small number of *PME* and *PMEI*s are strongly up-regulated upon germination, also in the presence of abscisic acid, which delays endosperm weakening and rupture [100]. Furthermore, overexpression of the PME inhibitor *PMEI5* resulted in a higher DM of seeds and reduced PME activity, which was accompanied by an earlier and faster germination process compared to wildtype [99]. This suggests that PMEI activity is essential for proper timing of seed germination but the precise molecular events are only beginning to be understood, based on modern techniques such as Chip-Seq [45] and potentially based on novel probes to dynamically monitor cell walls [101].

### 5.2. PMEI Function in Growth Processes

Plant cell growth is regulated by the interplay between the intracellular turgor pressure and the flexibility of the cell wall. Recent reviews have highlighted the importance of pectin, and especially HG, for mediating changes in the mechanical properties for control of growth processes [4,6,102]. In addition, studies using atomic force microscopy provided evidence that increased pectin de-methylesterification correlates with a decrease in cell wall stiffness [21,25].

A PMEI from *Arabidopsis*, AtPMEI4, has been shown to be involved in regulating hypocotyl growth. Growth acceleration of dark-grown hypocotyls requires significant cell wall remodeling, which is associated with transcriptional upregulation of pectin-modifying genes [103]. *AtPMEI4* was upregulated during this developmental transition and its expression was shown to be specific for rapidly elongating epidermis cells in hypocotyls and roots. In an overexpression line, the onset of hypocotyl growth acceleration was delayed, suggesting that the PME inhibitor controls pectin de-methylesterification essential for the timing of the growth acceleration, but not the growth process itself [103]. On the other hand, *OsPMEI28* overexpression in rice had an effect on the growth process, namely inhibition of culm elongation and decreased cell wall thickness of culms, which resulted in a dwarfed phenotype [61]. Conversely, a *pmei4* mutant with elevated PME activity in *Arabidopsis* root cell walls showed an increase in root length [62], suggesting a correlation between increased de-methylesterification and elevated growth.

Controversial results have been reported for purified kiwi PMEI applied on *Arabidopsis* root tips, where an increase of root growth rate was stated upon inhibition of PME activity [104]. Similarly, *Arabidopsis* plants overexpressing *AtPMEI2* show enhanced growth and increased biomass due to enhanced cell expansion [105] as well as increased root length due to stronger cell elongation [26]. Increased root length and area was also observed in *Arabidopsis* plants expressing a PMEI from cotton [39]. Therefore, higher levels of DM of pectin seem also to be able to promote growth and cell expansion.

This correlation was also observed in pollen tube growth. Pollen tubes undergo a fast and unidirectional growth at the apex dependent on ion (e.g., calcium) dynamics and PMEs have been shown to play a role in this process [106,107]. A pollen-specific PME inhibitor, AtPMEI2, was shown to inhibit PME activity in *Arabidopsis* [52]. Although the pollen-specific pectin methylesterase *AtPPME1* was expressed along the whole pollen tube, the localization of AtPMEI2 was exclusively restricted to the apex, the site of elongation. This supports the model that elongation requires higher DM of the cell wall, which is caused by inhibition of PME activity, specifically at the tip. Conversely, along the pollen tube, PME activity is less inhibited, resulting in de-methylesterification, which in turn would lead to formation of calcium cross-links due to the availability of free calcium ions, causing increased cell wall stiffness. Corroborative, expression of *AtPME1* in tobacco pollen tubes inhibited elongation, whereas *AtPMEI2* expression led to an increase in elongation rate [52]. Antisense expression of a pollen-specific *PMEI* from broccoli (*Brassica oleracea*) in *Arabidopsis* triggered silencing of the orthologous *Arabidopsis* gene At1g10770 and resulted in male sterility. This suggests that disturbance of PME activity imbalances the pectin distribution and affects cell wall dynamics in the pollen cell wall with detrimental effect on fertility [108]. In addition, wheat *Tdpmei2.1* and *Tdpmei2.2* transcripts have been shown to undergo complete mRNA processing, specifically in anthers [42]. The occurrence of this mechanism suggests a role of the PME inhibitors during pollen development and reproductive processes, but requires further investigations.

The present data indicate a complex relationship between changes of DM of pectin (based on PMEI activity) and growth processes. Further studies are necessary to elucidate in a tissue-specific manner how the interplay of PMEs and PMEIs regulate pectin methylesterification and affect mechanical properties of cell wall necessary for cell elongation.

### 5.3. PMEI Function in Organ Formation

The mechanical properties of cell walls have been proposed to play a key role in plant morphogenesis and the control of organ outgrowth [7,109,110]. Organ morphogenesis requires a transition from isotropic growth of undifferentiated cells to anisotropic growth pattern, which is dependent on local cell wall loosening and stiffening by e.g., modification of cell wall components.

*AtPMEI3*, which is expressed in the apical meristem, was inducible expressed in *Arabidopsis*, which led to hyper-methylesterification of HG throughout the meristem dome upon induction [24]. As a result, the formation of both floral and flower organ primordia was inhibited. Using atomic force microscopy, it was shown that the reduced de-methylesterification of HG was accompanied by increased stiffness of cell walls in the shoot apical meristem in the *AtPMEI3* overexpressing lines [25]. This further resulted in shorter cells and loss of growth asymmetry, showing that anisotropic growth requires an asymmetric loosening of longitudinal walls, which is caused by fine-tuned de-methylesterification of pectin.

Constitutive overexpression of *AtPMEI5* in *Arabidopsis* also caused strong morphological phenotypes [111,112]. The stems of the transgenic plants showed an aberrant, twisted growth pattern, especially at branching points and branching organs (e.g., leaf or inflorescence), which failed to separate properly from the main stem [111]. Additionally, root growth was disturbed in the mutant and curled leaves and misshapen siliques were observed, which was proposed to be a result of activated BR signaling [112]. This indicates that PMEIs are involved in the fine-tuning of cell wall biomechanical properties via HG methylesterification that is required for proper organ formation and separation.

PMEIs might also play a role in organ senescence as changes in pectin methylesterification have been described, e.g., for petal senescence [113,114]. However, so far, only transcriptional changes of putative invertase/pectin methylesterase inhibitor genes in petals during senescence have been reported and further, more detailed studies will be necessary to investigate their putative function.

### 5.4. PMEIs in Fruit Development

Fruit ripening is a highly controlled developmental event during which the fruit undergoes various changes, e.g., in sugar contents, color, flavor, aroma, texture. Softening is a very (economically) important physiological change and several cell wall-modifying enzymes are involved in this process [115]. The first inhibitor of PME was identified in ripe kiwi fruit by Balisteri et al. [33] and was found active only in the mature stage, whereas it was undetectable in unripe fruits [116]. Furthermore, the expression levels of the *PMEI* genes in kiwi fruit increased with the progression of fruit maturation [117]. In banana (*Musa acuminata*), a PME inhibitor, *MaPMEI1*, was shown to be specifically expressed in the fruit and up-regulated during the ethylene-dependent ripening process [46]. Similarly, a PMEI has been identified in the tomato fruit [59]. *SolyPMEI* was strongly expressed in red fruits and, in lower levels, also in flowers and pollen, but not in green fruits. Interaction with the tomato PME-1 enzyme in the fruit and increased *SolyPMEI* expression levels with progression of ripening stages implicates the inhibitor in modulating PME activity and degree of methyl-esterification during fruit ripening in tomato. In grape (*Vitis vinifera*), *VvPMEI1* was found to be expressed only during the early phases of berry development, characterized by a rapid berry growth both through cell division and expansion [41]. *VvPMEI1* could be involved in regulating PME activity required for rapid cell growth and to maintain firmness, or it could prevent early softening of grape berries related to pectin degradation. This indicates a role for PMEIs in modulating PME activity and pectin methylesterification in different stages of fruit development.

## 6. Potential of PMEIs for Applications

Lignocellulosic biomass constitutes a sustainable renewable source of chemicals and fuels, but a major bottleneck for an industrial utilization is the natural recalcitrance of the cell wall due to its complex and heterogeneous structure. Modification of cell wall polysaccharides can improve the degradation into soluble sugars, which can be used by microorganisms for fermentation, a process called saccharification [118]. Improved saccharification can make an industrial process more efficient and reduce cost for pre-treatments of the biomass. Lionetti et al. [105] showed that overexpression of *AtPMEI2* in *Arabidopsis* leads to a higher enzymatic saccharification efficiency, indicating a reduced cell wall recalcitrance likely due to the higher degree of HG methylesterification. The same was observed for wheat plants expressing the kiwi *PMEI*, suggesting that both cellulose and hemicellulose are better digestible during enzymatic hydrolysis in the transgenic plants. As a side effect, transgenic *Arabidopsis* plants had a higher biomass yield due to enhanced cell expansion. Further, a negative correlation between the level of de-methylesterified HG and cellulose degradability was found for 24 *Arabidopsis* accessions [119]. The reduction of egg-box structures or other crosslinks of methylesterified HG might be responsible for the accessibility of cellulose fibers for enzymatic digestion, which in turn results in increased saccharification efficiency. This indicates the potential of PMEI as a HG modifying enzyme to improve plant tissues for saccharification by targeted mutation or in wide-scale selection and breeding of plants for further utilization (e.g., as biofuels or for production of chemicals).

PMEIs have been shown to play a role in ripening processes (see above). Therefore, a potential application of this inhibitor would be in food (fruit and vegetable) processing. For food technology, endogenous PME activity is highly relevant because it determines the physiochemical and functional properties of pectin, thereby affecting the quality of plant-derived food products. PME activity in fruit juice causes undesired phase separation because de-methylesterified HG forms calcium crosslinks, and the resulting calcium pectate is insoluble and precipitates [120]. Addition of PMEI to orange juice was shown to control PME activity even over long storage, thereby avoiding phase separation. Application of PMEI would reduce required thermal treatments during juice processing and should result in better flavor and product quality [120]. For development or optimization of food-processing steps, it would be beneficial to determine PME activity in plant tissues. A PMEI-based molecular probe was shown to successfully label plant PMEs in various plant tissues and moreover, to differentiate between active and inactivated PME molecules [121]. This application of PMEIs would give important information about presence and localization of active PMEs after different food processing steps (e.g., thermal or high pressure treatment, microwaving). PMEIs have also been proposed for application in wine and marc production [41]. Pectin de-methylesterification releases high levels of methanol during different stages of grape processing, greatly affecting the composition of the final product [122,123]. Here, PMEIs could be used to reduce methanol formation in grape must and marc, as well as in products derived by fermentation and distillation [124]. Apart from applications in food industry, manipulation of PMEIs in planta could also be of interest. Overexpression of *PMEI*s, in banana fruits for example, would not only control over-softening but also likely extend banana shelf-life [46]. In conclusion, PMEIs could be good tools to control endogenous PME activity during food production processes, thereby positively influencing product quality.

As mentioned above, PMEIs play an important role in the response to biotic and environmental stresses. Therefore, the inhibitors could be potential targets for genetic engineering, aimed to improve plant fitness. In cold as well as salt stress conditions, PMEIs might be beneficial for balancing growth maintenance with stress tolerance and overexpression of *PMEI*s would be a promising way to improve salt tolerance of crops [89]. In addition, PMEI activity has been shown to be essential for resistance against fungal and bacterial pathogens [26,79,83]. Therefore, it could be useful as a potential molecular marker in plant breeding programs targeted to the selection of crop varieties with durable resistance.

## Abbreviations

PMEI	Pectin methylesterase inhibitor
PME	Pectin methyl esterase
DM	Degree of methylation
HG	Homogalacturonan
GalA	D-galacturonic acid
GAUT	Galacturonosyltransferases
OGs	Oligogalacturonides
DAMP	Damage associated molecular pattern
H_2_O_2_	Hydrogen peroxide
SCE	Seed coat epidermis
MUM1	Mucilage-modified mutant 1
